# Non‐coplanar CBCT image reconstruction using a generative adversarial network for non‐coplanar radiotherapy

**DOI:** 10.1002/acm2.14487

**Published:** 2024-08-26

**Authors:** Ran Wei, Zhiyue Song, Ziqi Pan, Ying Cao, Yongli Song, Jianrong Dai

**Affiliations:** ^1^ Department of Radiation Oncology, National Cancer Center/National Clinical Research Center for Cancer/Cancer Hospital Chinese Academy of Medical Sciences and Peking Union Medical College Beijing China

**Keywords:** cone‐beam computed tomography, deep learning, image reconstruction, limited angle, non‐coplanar radiotherapy

## Abstract

**Purpose:**

To develop a non‐coplanar cone‐beam computed tomography (CBCT) image reconstruction method using projections within a limited angle range for non‐coplanar radiotherapy.

**Methods:**

A generative adversarial network (GAN) was utilized to reconstruct non‐coplanar CBCT images. Data from 40 patients with brain tumors and two head phantoms were used in this study. In the training stage, the generator of the GAN used coplanar CBCT and non‐coplanar projections as the input, and an encoder with a dual‐branch structure was utilized to extract features from the coplanar CBCT and non‐coplanar projections separately. Non‐coplanar CBCT images were then reconstructed using a decoder by combining the extracted features. To improve the reconstruction accuracy of the image details, the generator was adversarially trained using a patch‐based convolutional neural network as the discriminator. A newly designed joint loss was used to improve the global structure consistency rather than the conventional GAN loss. The proposed model was evaluated using data from eight patients and two phantoms at four couch angles (±45°, ±90°) that are most commonly used for brain non‐coplanar radiotherapy in our department. The reconstructed accuracy was evaluated by calculating the root mean square error (RMSE) and an overall registration error *ε*, computed by integrating the rigid transformation parameters.

**Results:**

In both patient data and phantom data studies, the qualitative and quantitative metrics results indicated that ± 45° couch angle models performed better than ±90° couch angle models and had statistical differences. In the patient data study, the mean RMSE and *ε* values of couch angle at 45°, −45°, 90°, and −90° were 58.5 HU and 0.42 mm, 56.8 HU and 0.41 mm, 73.6 HU and 0.48 mm, and 65.3 HU and 0.46 mm, respectively. In the phantom data study, the mean RMSE and *ε* values of couch angle at 45°, −45°, 90°, and −90° were 91.2 HU and 0.46 mm, 95.0 HU and 0.45 mm, 114.6 HU and 0.58 mm, and 102.9 HU and 0.52 mm, respectively.

**Conclusions:**

The results show that the reconstructed non‐coplanar CBCT images can potentially enable intra‐treatment three‐dimensional position verification for non‐coplanar radiotherapy.

## 1 INTRODUCTION

Non‐coplanar radiotherapy with couch rotation is commonly used in stereotactic radiotherapy and stereotactic body radiotherapy.^1^, ^2^, ^3^ However, couch rotation increases the treatment time, which can increase the magnitude and probability of patient intrafraction movement.^4^, ^5^, ^6^ The position errors introduced after couch rotation can reach up to 2 mm.^7^ Besides the position errors introduced by the couch rotation, it is reported that patient intrafraction movement within the frameless thermoplastic mask may be larger than 5 mm.^8^ Ongoing position verification during treatment is vital to correct any patient intra‐fractional movement.

Optical Surface Imaging (OSI) systems, such as Catalyst HD, Optical Surface Monitoring System, and so forth, are popularly used for patient position verification and monitoring, due to the following desirable characteristics: imaging without dose, real‐time monitoring, and so forth. However, some studies have also pointed out that there are some problems with the OSI system in verifying patients' positions.^9^, ^10^, ^11^, ^12^, ^13^ Lai et al.^9^ directly compared the position accuracy between Catalyst HD and cone‐beam computed tomography (CBCT) regarding the non‐coplanar setup in brain patients. They found poor agreement between the two systems and concluded that Catalyst HD may not replace CBCT. Some studies also found that OSI systems may report false offsets during the monitoring of stationary phantoms.^11^, ^12^ Covington et al.^13^ found that ongoing improvements in software and calibration procedures of OSI systems can decrease the reporting of false offsets, while the false offset magnitude for non‐coplanar situations is still larger than that for coplanar situations. Therefore, it is recommended to use x‐ray images (such as CBCT) to re‐image the patient to adjust their positions when OSI systems observe an offset exceeding the tolerance.^13^, ^14^

Owing to the possible collision of the gantry and treatment couch, it is challenging to apply CBCT to verify the patient's position in non‐coplanar situations.^2^ Conventional linear accelerators require projections covering a scanning range of at least 200° to reconstruct CBCT images.^15^ In non‐coplanar radiotherapy, the scanning range is less than 200° at certain couch angles, and the number of projections obtained is thus limited.^16^ The CBCT image cannot be reconstructed from limited‐angle projections or the reconstructed CBCT image has artifacts (streaks and/or edge distortions) using conventional reconstruction algorithms, such as the Feldkamp–Davis–Kress (FDK) algorithm.^17^, ^18^, ^19^, ^20^

Previous studies have developed iterative methods to reconstruct CBCT images using limited‐angle projections. Zeng et al.^21^ applied a total variation constraint to remove artifacts and mitigate noise in limited‐angle CBCT images. Xu et al.^22^ proposed a CBCT image reconstruction algorithm based on guided image filtering, which can transfer the features of the guidance image to the target image and help determine the iterative direction. Donoho proposed the theory of compressed sensing (CS), which provides a new way to solve the reconstruction problem of limited‐angle CBCT images.^23^ Chen et al.^24^ introduced the prior image constraint on the CS algorithm (PICCS) to improve the quality of reconstructed images. Meng et al.^16^ improved PICCS by using registration to update successively the prior image to deal with the invalidity of PICCS in situations in which the prior image deviates significantly from the reconstructed image. However, these iterative methods have the potential drawbacks of prolonged reconstruction time and high computational costs, and their reconstruction performance is affected by the iteration parameters.^25^, ^26^

Recently, owing to the outcomes of deep learning in computer vision and image processing, some studies have used deep learning for CBCT image reconstruction with limited‐angle projections.^27^, ^28^, ^29^, ^30^, ^31^, ^32^ Würfl et al.^27^ mapped filtered back‐projection‐type algorithms to neural networks and proposed a new type of cone‐beam back‐projection layer to reconstruct CBCT images using projections with the scanning range limited to 180°. Dai et al.^28^ trained a model to generate the missing parts of full‐view projections using three‐dimensional (3D) sinogram images of limited‐angle projections, after which the CBCT images were reconstructed from the generated full‐view projections using the FDK algorithm. Zhang et al.^29^ proposed an unsupervised two‐dimensional (2D)−3D deformable registration network (2D3D‐RegNet) to reconstruct CBCT images within 5 s using projections orthogonally arranged covering a scanning range of 90°. Lu et al.^30^ proposed a geometry‐guided model using projections limited to 199°. Hu et al.^31^ developed the structure‐enhanced attention network (SEA‐Net) method for limited‐angle CBCT image reconstruction. The proposed SEA‐Net performs well for scanning ranges larger than 135°. Although these networks exhibit satisfactory performance in high‐quality CBCT image reconstruction using limited‐angle projections, they are all reconstruction methods for coplanar situations and may encounter a challenge when used for non‐coplanar CBCT image reconstruction: the available scanning range is greatly reduced in non‐coplanar radiotherapy owing to the possible collisions between the gantry, couch, patient, and onboard imaging system.

The purpose of this study is to develop a robust non‐coplanar CBCT image reconstruction model, trained using coplanar CBCT as prior information and non‐coplanar limited‐angle projections to reconstruct high‐quality non‐coplanar CBCT images. To the best of our knowledge, this is the first study that focuses on non‐coplanar CBCT image reconstruction using deep learning.

## 2 MATERIALS AND METHODS

### 2.1 Data acquisition and preprocessing

#### 2.1.1 Patient data acquisition

Forty patients with brain tumors were retrospectively included. One coplanar CBCT scan was acquired using the FDK algorithm configured in the onboard imaging system on a Varian TrueBeam linear accelerator (Varian Medical Systems, Inc., Palo Alto, CA, USA) in full‐fan mode was acquired for each patient before initial treatment.^33^ The x‐ray tube was operated at 100 kV and 15 mA with the pulse width at each projection angle of 20 ms. The reconstructed coplanar CBCT size was 512 × 512 × 93 voxels with a spacing of 0.5112 mm × 0.5112 mm × 1.99 mm.

#### 2.1.2 Patient data preprocessing

The patient data, such as the ground truth and limited‐angle non‐coplanar projections, were simulated in the data preprocessing step. First, the coplanar CBCT image of each patient was rotated by the four couch angles listed in Table 1 to simulate patient setup in non‐coplanar radiotherapy. These four couch angles (±45°, ±90°) were selected because they are the most commonly used in non‐coplanar radiotherapy in our department. The non‐coplanar images of each patient were then randomly moved to introduce rotation errors (range: −3° to 3°) and translation errors (range: −10 to 10 mm) from the original position in one or more axes or directions to simulate patient setup errors under realistic conditions. The random sampling of translation and rotation both follow a uniform distribution. The minimum interval of random sampling for translation and rotation were 0.1 mm and 0.1°, respectively. The ranges of introduced errors were chosen in accordance with the linear accelerator's changeable pitch and roll angle ranges (0°−3°) and more sophisticated translation conditions used in other study.^34^ In this study, the error‐introduced step and ray‐casting step would be performed five times for each non‐coplanar image, which would increase the size of datasets. The augmented non‐coplanar CBCT images associated with the setup errors would be used as the ground truth in the patient data study. Subsequently, a ray‐casting algorithm was used to generate digitally reconstructed radiographs (DRR) with a limited scanning range to simulate limited‐angle projections obtained at non‐coplanar couch angles.^35^ The projection range depended on the couch angle and is listed in Table 1. The source‐to‐skin distance was 1000 mm, and the source‐to‐image detector distance was 1500 mm. The projection size was 1024 × 768 pixels, the pixel size was 0.388 mm × 0.388 mm, and the projection frequency was 0.4°/frame. These parameters were the same as those used in the projection acquisition of coplanar CBCT images. Among these parameters, the value of the projection frequency is set by the TrueBeam system and is not changeable.^36^

**TABLE 1:** The general information of couch angles, projection range, and projection numbers to simulate non‐coplanar radiotherapy.

Couch angle (°)	Projection range	Number of projections
45	180°−330°	375
−45	330°−120°	375
90	250°−310°	150
−90	30°−120°	225

#### 2.1.3 Phantom data

**FIGURE 1:**
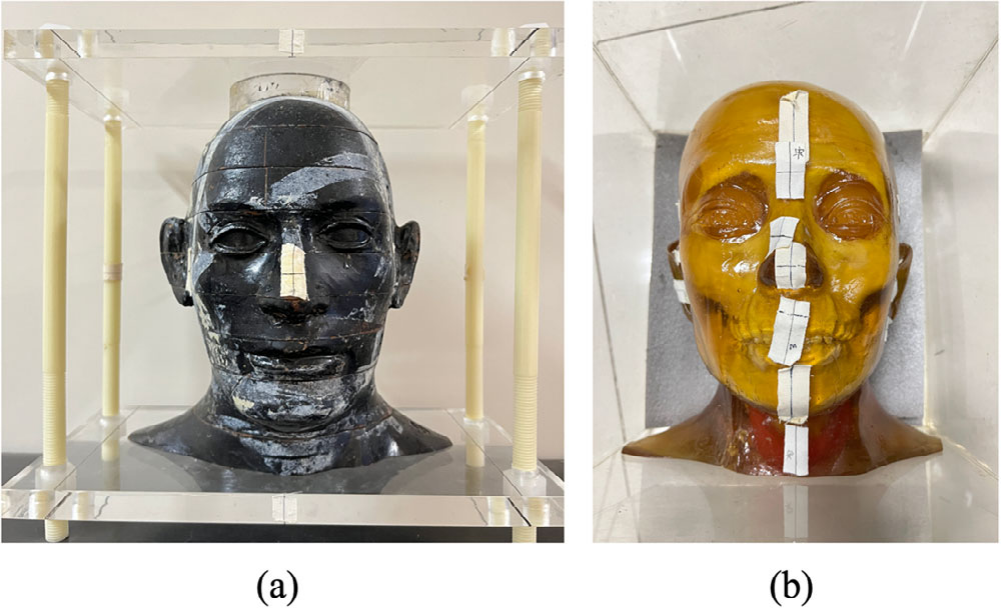
Two head phantoms were used for model evaluation. (a) Alderson–Rando Head Phantom. (b) Head‐neck anthropomorphic phantom.

Two anthropomorphic head phantoms (Radiological Support Services, Long Beach, CA, USA) were used in this study (Figure 1a and b). The phantom was positioned at the isocenter position using the room lasers, after which the first coplanar CBCT image was acquired. The scanning parameters of coplanar CBCT were the same as those used for the patient data. The phantom was then deliberately positioned away from the isocenter position to simulate setup errors (rotational error range: −3° to 3°, translational error range: −10 to 10 mm), and the second coplanar CBCT image was then acquired. The couch was then successively rotated to the four couch angles listed in Table 1, and four sets of non‐coplanar projections were acquired for each phantom. This step was repeated three times for each phantom. During each repeated operation, the phantoms were positioned first with different random setup errors, and then the second coplanar CBCT images and non‐coplanar projections at four couch angles were acquired. It is noted that the accuracy of treatment couch movement (including translation and rotation) had been verified before measurements.^37^ The projection parameters were the same as those of the patient data. Given the challenge of reconstructing non‐coplanar CBCT images on the current linear accelerator, the second coplanar CBCT images were rotated according to the four rotation angles listed in Table 1 and served as ground‐truth CBCT (gCBCT) images.^33^, ^38^

### 2.2 Network structures

**FIGURE 2:**
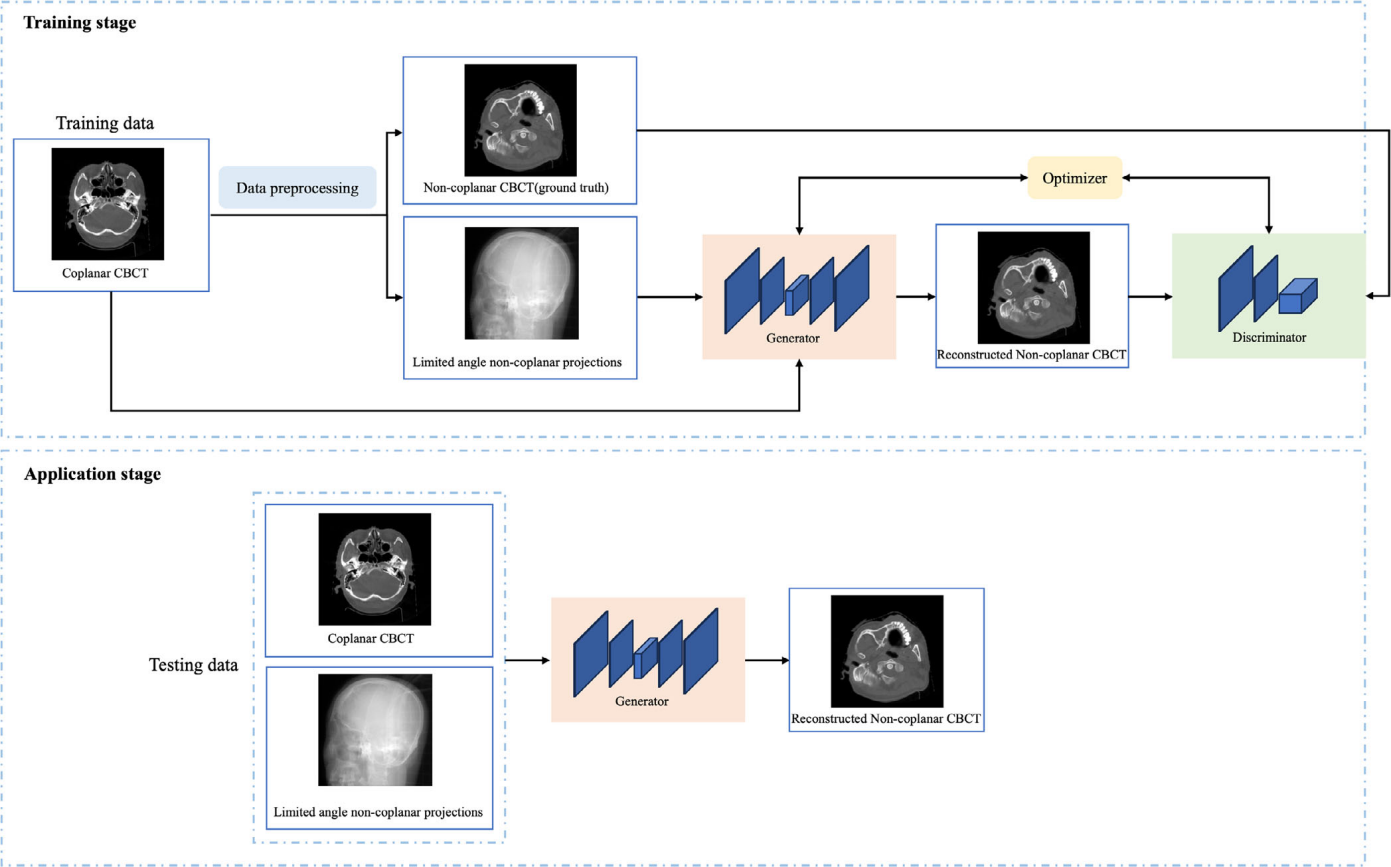
The overall structure of the non‐coplanar CBCT image reconstruction method. CBCT, cone‐beam computed tomography.

As shown in Figure 2, the proposed method comprises two stages: training and application. In the training stage, the coplanar CBCT image and the limited‐angle non‐coplanar projections were fed into a generative adversarial network (GAN) model, which contained a generator and a discriminator.^18^, ^39^, ^40^, ^41^, ^42^, ^43^ The numbers of the limited‐angle non‐coplanar projections depended on the couch angle and are listed in Table 1. The generator was trained to produce a reconstructed non‐coplanar CBCT (rCBCT) image that could not be distinguished from the gCBCT image, whereas the discriminator was adversarially trained to detect the rCBCT image from the generator. In the application stage, the coplanar CBCT image and non‐coplanar CBCT projections were input into the trained generator of the GAN model, and a non‐coplanar CBCT image was acquired. Four GAN models were trained according to the four couch angles listed in Table 1. The GAN model is the variant of the one we used before and details are introduced below.^44^

**FIGURE 3:**
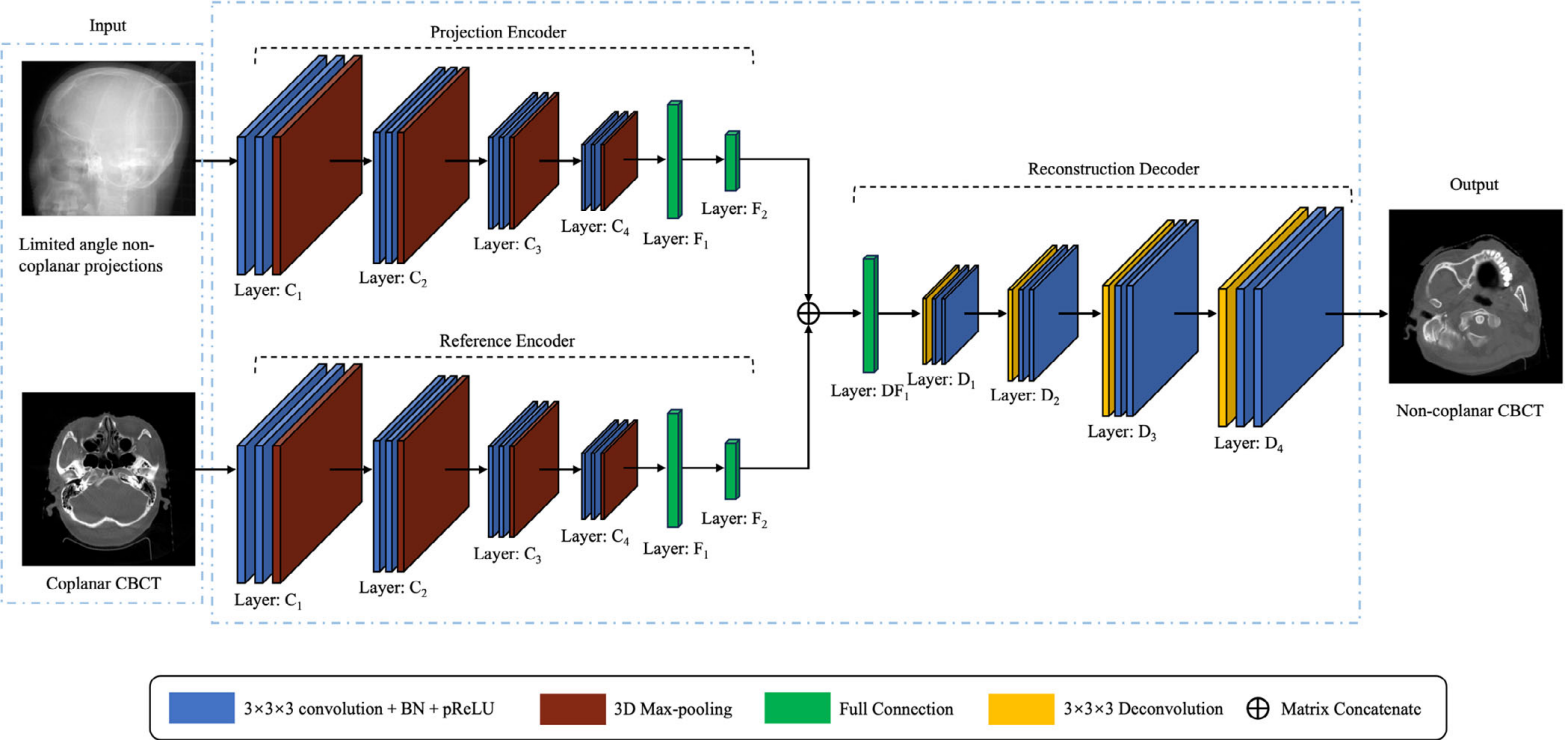
The architecture of the generator.

The architecture of the generator, which is based on an encoder‐decoder structure, is shown in Figure 3. Each encoder contains four convolutional blocks and two fully connected layers, whereas the decoder contains a fully connected layer and four deconvolutional blocks. Each convolutional block contains two 3 × 3 × 3 convolutional layers with a stride of 2 × 2 × 2 and a max‐pooling layer. Following each 3 × 3 × 3 convolutional layer, there is a batch normalization (BN) layer and a parametric leaky rectified linear unit (pReLU) layer (i.e., 3 × 3 × 3 convolution+BN+pReLU). Similarly, each deconvolutional block contains a deconvolutional layer with a stride of 2 × 2 × 2 followed by two 3 × 3 × 3 convolution+BN+pReLU layers.

**FIGURE 4:**
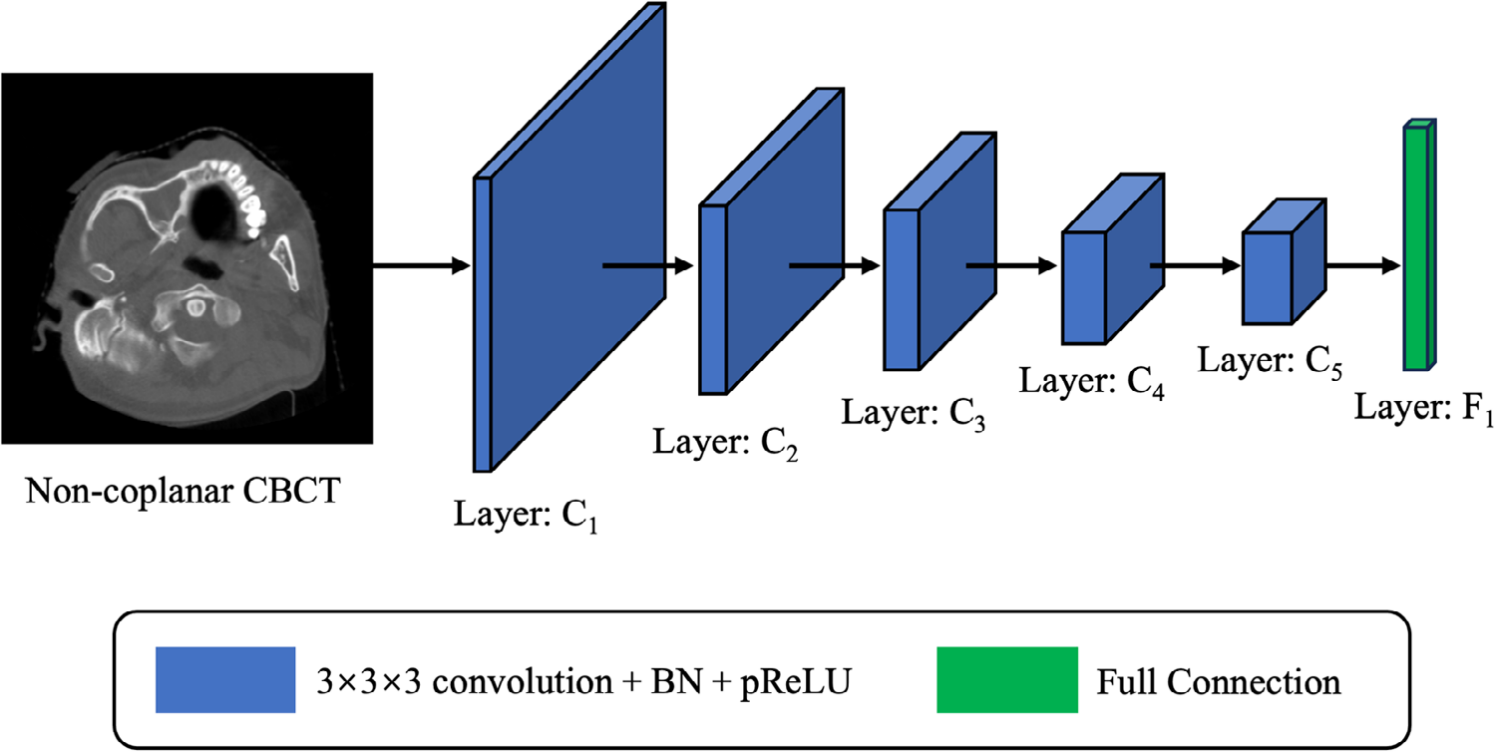
The architecture of the discriminator.

The architecture of the discriminator is shown in Figure 4, which shows the patch‐based convolutional neural network structure with five 3 × 3 × 3 convolution+BN+pReLU layers and a full connection layer. The input of the discriminator only requires true or fake non‐coplanar CBCT image (i.e., gCBCT image or rCBCT image), and the output is generated using a sigmoid function in the last fully connection layer.

### 2.3 Loss function design

In this study, we propose a new joint loss function to train the network. The joint loss function is expressed as:

(1) $$\begin{array}{ccc} L\; & = & \;arg\min_{G}\max_{D}L_{GAN}\left(G,D\right)\\ & & +\;\lambda 1L_{L1}\left(G\right)++\lambda 2L_{Rigid}\left(G\right) \end{array}$$

where G is the generator, and D is the discriminator. $L_{GAN}\left(G,D\right)$ is a conventional GAN loss and is calculated as follows,

(2) $$\begin{array}{ccc} L_{GAN}\left(G,D\right)\; & = & E_{gCBCT,\;iData}\;\left[logD\left(gCBCT\right)\right]\\ & & E_{iData}\left[logD\left(G\left(iData\right)\right)\right] \end{array}$$

Here iData represents the input data, which includes the coplanar CBCT image and non‐coplanar CBCT projections.

The $L_{L1}\left(G\right)$ term in Equation (1) refers to the L1‐norsm loss between the ground truth and the reconstructed one. The term λ1 is the weight of the L1‐norm loss, which is empirically set to be 100 in this study.^44^, ^45^

(3) $$L_{L1}\left(G\right)\;=E_{gCBCT,iData}\;\left[{∥gCBCT-G\left(iData\right)∥}_{1}\right]$$

$L_{Rigid}\left(G\right)$ represents the rigid‐bone loss function that we defined to improve the bone structure consistency between the rCBCT image and coplanar CBCT image. Some studies have applied structural consistency terms, such as SSIM, in the loss function to reduce the anatomy deformation in the reconstructed image.^27^, ^28^, ^30^, ^31^, ^32^, ^33^, ^34^, ^46^ However, these losses cannot achieve global structure consistency for certain components of the patient.^47^ For example, the deformations in the bone structure in the images acquired during brain radiotherapy were minor, while deformation may be generated in the oral cavity and nasal cavity due to the existence of swallowing or breathing. In addition, there is a large rotational deviation between the coplanar CBCT image and the non‐coplanar CBCT image due to the rotation of the treatment couch. Thus, we proposed the rigid‐bone loss to improve the global structure consistency between the rCBCT image and the coplanar CBCT image. The $L_{Rigid}\left(G\right)$ could be expressed as,

(4) $$\begin{array}{ccc} L_{Rigid}\;\left(G\right) & = & E_{cCBCT,iData}\left[∥CM\left(Edge\left(Bone\left(cCBCT\right)\right)\right)\right.\\ & & \left.-\;CM\left(Edge\left(Bone\left(G\left(iData\right)\right)\right)\right){∥}_{2}\right] \end{array}$$

In this case, cCBCT represents the coplanar CBCT image, and Bone(·) was a function that extracted the bone structures of the input image. In general, bone structures can be extracted by setting a threshold that sets the pixel values of other unnecessary structures to zero. However, this operation is not differentiable, which would make the calculation of the gradient of the loss function impossible. Thus, we utilized the following sigmoid function for bone extraction,

(5) $$Bone\;\left(I\right)=\;Sigmoid\left(Scale*\left(I-BoneThreshold\right)\right)$$

where, I is the input image that refers to the coplanar CBCT image or the reconstructed noncoplanar CBCT image. BoneThreshold stands for the threshold for bone, which is empirically set to 1000 HU.^48^ Scale is a large scaling factor, which makes the slope in the Sigmoid function more steep and is set to be 100000 in this study. Edge(·) was an edge extraction function that utilized the Sobel kernel.^49^ As the edge extraction was essentially based on convolution operation, the gradient can be calculated. CM(·) represents the function that computed the central moment of the input data.^50^ As there are rotations for the bone structure, we utilized the central moment that has translation and rotation invariance to improve the consistency of bone structure. It needs to be clear the CM(·) does not output a scalar but a k‐dimensional vector. The k‐th element of the output from CM(·) refers to the k‐order central moment of the input data, which was set to be four in this study. ${∥\cdot ∥}_{2}$ was the function that calculated the L2 norm of the input vector. The term λ2 is the weight of the L2‐norm loss, which was set to be 0.1 in this study.^51^

### 2.4 Model training and evaluation

In this study, 40 coplanar CBCT scans and 800 sets of non‐coplanar projections (40 patients, four couch angles, repeated five times) were included in the patient data set. In the phantom data set, two coplanar CBCT images (only the first coplanar CBCT images were used here) and 24 sets of non‐coplanar projections (two phantoms, four couch angles, repeated three times) were included. For network training, 80% of the patient data (data from 32 patients) were randomly selected as the training dataset, whereas the remaining 20% of the patient data and the phantom data were used as the testing dataset to evaluate the performance of the trained model based on both qualitative and quantitative analyses.

The network was established with the Pytorch framework and trained with a workstation that has a CPU of Intel i9‐13900K, a GPU of NVIDIA RTX 4090, and 64 GB RAM. The learning rate for network training was initially set to be 0.002 and decreased by 30% for each epoch. The training was terminated if the mean square error between the synthetic DRR and DRR showed a variation below 5% for more than three epochs. The whole training process took 98 epochs, which lasted for approximately 3 days.

#### 2.4.1 Qualitative analysis

(6) $$Image_{diff}=\;gCBCT-rCBCT$$

The difference image ($Image_{diff}$) is plotted using the value between the gCBCT and the rCBCT.

#### 2.4.2 Quantitative analysis

(a) Root Mean Square Error (RMSE)

(7) $$RMSE\;=\sqrt{\sum_{i,j,k}rCBCT_{i,j,k}-gCBCT_{i,j,k}}\;$$

where i, j, and k are the position indices for the pixel in the gCBCT image and the rCBCT image.

(b) Overall Registration Error

(8)

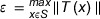

where T is the rigid transformation matrix between the rCBCT image and the gCBCT image, which is calculated using the SlicerANTs module in the 3D slicer (ver.5.0.3).^52^, ^53^ x represents the coordinates of the points on the surface of a sphere (S) with a center at the origin, a radius of 30 mm, and an angular separation of 5°.^54^, ^55^

## 3 RESULTS

### 3.1 Evaluation using patient data

**FIGURE 5:**
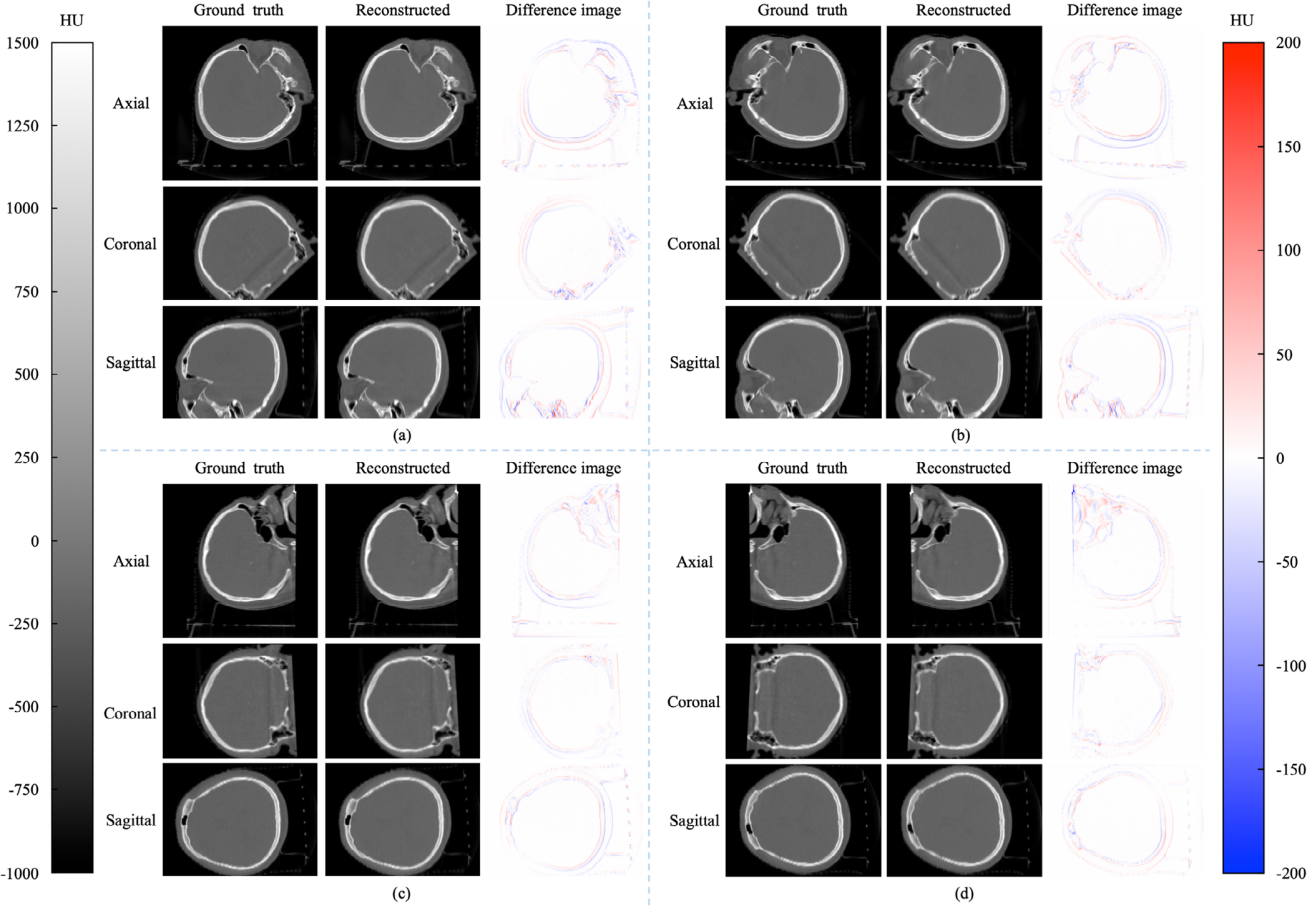
The reconstructed and ground truth central slices for patient 1 in the axial, coronal, and sagittal planes for couch angles of (a) 45°, (b) −45°, (c) 90°, and (d) −90°. Color bars on both sides indicate the image display window/level.

**FIGURE 6:**
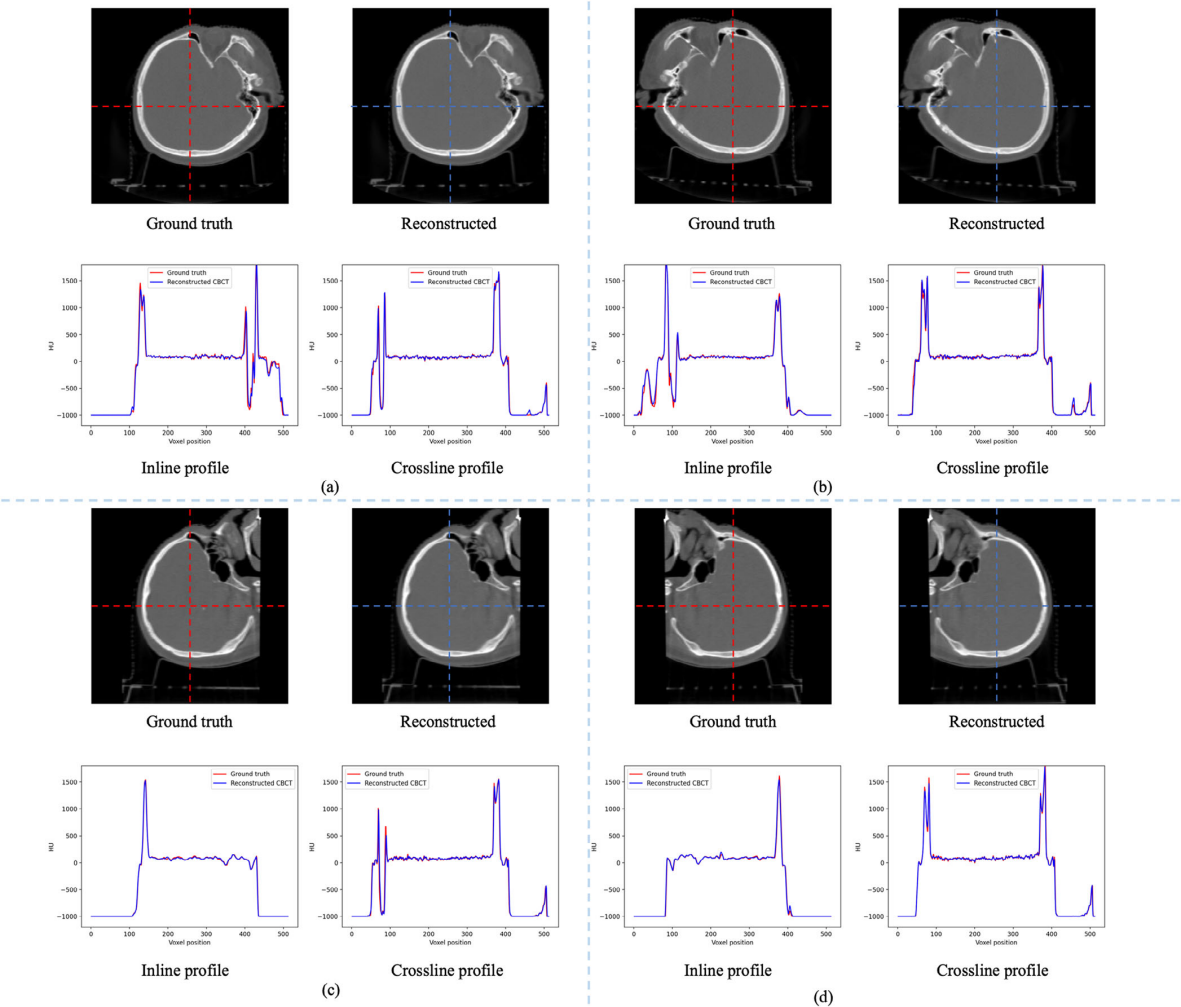
The inline and crossline of the axial plane of the central slice of the gCBCT and rCBCT images for patient 1 for couch angles of (a) 45°, (b) −45°, (c) 90°, and (d) −90°. The positions of the inline and crossline profiles are marked by the red and blue dashed lines, respectively. rCBCT, reconstructed non‐coplanar CBCT.

Figure 5 illustrates the central slices of the rCBCT and gCBCT images in the axial, coronal, and sagittal planes at the four couch angles. Difference images are also shown in Figure 5 illustrates the efficacy of the proposed method. It is clear that our method faithfully maintains the anatomical structures in the rCBCT images, which have high consistency with the gCBCT images in the four couch angle cases. The reconstructed images at the four couch angles yield less prominent blurring artifacts and lower inaccuracy, as demonstrated by the difference images. This finding applies to the profiles as well, as illustrated in Figure 6. The inline and crossline profiles show good agreement between the gCBCT and rCBCT images in the four couch angles cases.

**FIGURE 7:**
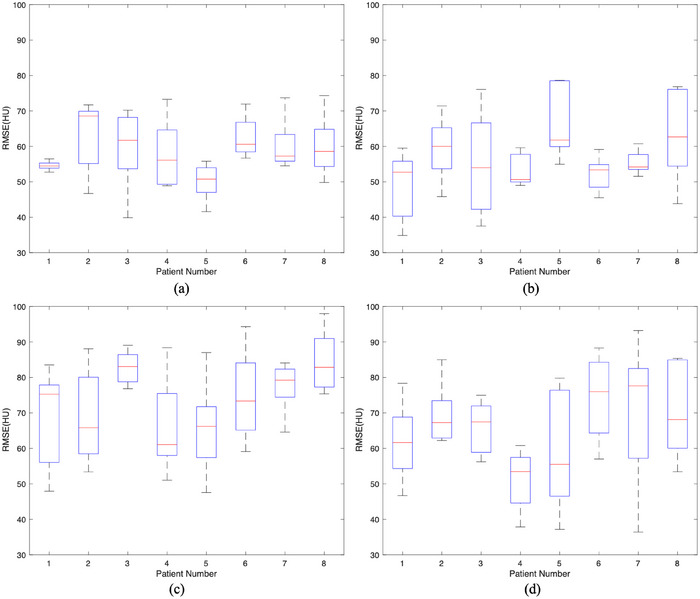
The RMSE between gCBCT and rCBCT images using patient testing data for couch angles of (a) 45°, (b) −45°, (c) 90° and (d) −90°. rCBCT, reconstructed non‐coplanar CBCT; RMSE, root mean square error.

Figure 7 shows boxplots of RMSE values comparing the gCBCT and rCBCT images in the cases of the four couch angles. The largest RMSE values were 74.32 HU, 78.61 HU, 98.01 HU, and 93.19 HU for the couch angles of (a) 45°, (b) −45°, (c) 90°, and (d) −90°, respectively. The mean RMSE values were 58.49 HU, 56.82 HU, 73.63 HU, and 65.34 HU for the couch angles of (a) 45°, (b) −45°, (c) 90°, and (d) −90°, respectively. There were no statistical differences in the comparisons of RMSE values between the group couch angles 45°and −45° (*p* = 0.8638), while there were statistical differences in the comparisons of RMSE values between the others.

**FIGURE 8:**
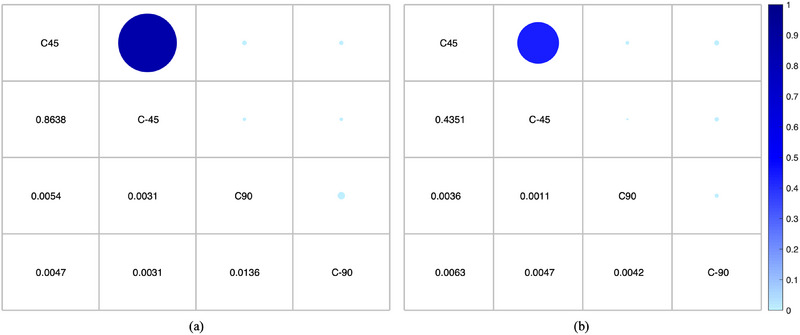
The *p*‐values of (a) the RMSE value and (b) the ε value compared multiple times under four couch angle groups in patient data evaluation. C45, C‐45, C90, and C‐90 represent the couch angle of 45°, −45°, 90°, −90°, respectively. The larger the *p*‐value, the larger the corresponding blue circle and the darker the color. RMSE, root mean square error.

The reconstructive quality was also quantitatively evaluated using the overall registration error ε (Table 2). The registration error increased when the scanning range was reduced, which reduced the number of projections. Moreover, given the same number of projections, the couch 45° group and couch −45° group share similar *ε* results and have no statistical difference (*p* = 0.4351) as shown in Figure 8b. The ε value for the couch 90° group was the largest among the four couch angle groups, whereas that for the couch −45° group was the smallest. The introduced setup errors could be detected in the reconstructed CBCT images and the ε values for all couch groups were within 1 mm.

**TABLE 2:** The transformation parameters (mean ± std) and overall registration error (mean ± std) between gCBCT and rCBCT patient testing data at four couch angles.

Couch angle (°)	$t_{x}$ (mm)	$t_{y}$ (mm)	$t_{z}$ (mm)	$r_{x}$ (°)	$r_{y}$ (°)	$r_{z}$ (°)	ε (mm)
45	0.164 (0.068)	0.228 (0.054)	0.222 (0.052)	0.181 (0.041)	0.146 (0.032)	0.143 (0.032)	0.417 (0.107)
−45	0.154 (0.070)	0.212 (0.058)	0.227 (0.067)	0.194 (0.038)	0.158 (0.030)	0.154 (0.030)	0.414 (0.119)
90	0.193 (0.083)	0.243 (0.064)	0.259 (0.061)	0.232 (0.060)	0.178 (0.066)	0.194 (0.092)	0.480 (0.155)
−90	0.207 (0.086)	0.232 (0.056)	0.245 (0.054)	0.226 (0.052)	0.182 (0.064)	0.197 (0.084)	0.461 (0.150)

In addition, we analyzed the effects of the rigid‐bone loss on the performance of the proposed method. First, it was analyzed whether the rigid‐bone would affect the speed of convergence of training loss. As shown in Figure 13a, there is not much difference between training loss values with and without the rigid‐bone loss. This may be because the GAN loss took account for the major part of the total loss. Actually, in the training process, to achieve the best performance of GAN, we adjusted the learning rate so that the generator and discriminator were well‐matched in strength. In this way, the generator always tried to output synthetic images that the discriminator could not distinguish and the discriminator always attempted to find fake images, which made the GAN loss stay at a relatively large level during the whole training process. Thus, rigid‐bone loss won't have a major impact on the speed of convergence of the training loss. On the other hand, we analyzed the efficacy of the rigid‐bone loss with SSIM. On the ground truth as well as results with and without rigid‐bone loss, we utilized a threshold of 1000 HU to extract bone structures. Then the SSIM value for bone structures was calculated. Figure 13b demonstrates the SSIM values for bone structures with and without the rigid‐bone loss. It can be seen, in spite of the fluctuation, the SSIM values with rigid‐bone loss are higher than those without rigid‐bone loss. Figure 14 demonstrates an example of the reconstruction results with and without rigid‐bone loss of patient 1. It can be seen the result without rigid‐bone loss had distortion in bone structure (red dashed line box), while the result with rigid‐bone loss was highly consistent with the ground truth. These results implied the effectiveness of the rigid‐bone loss.

### 3.2 Evaluation using phantom data

**FIGURE 9:**
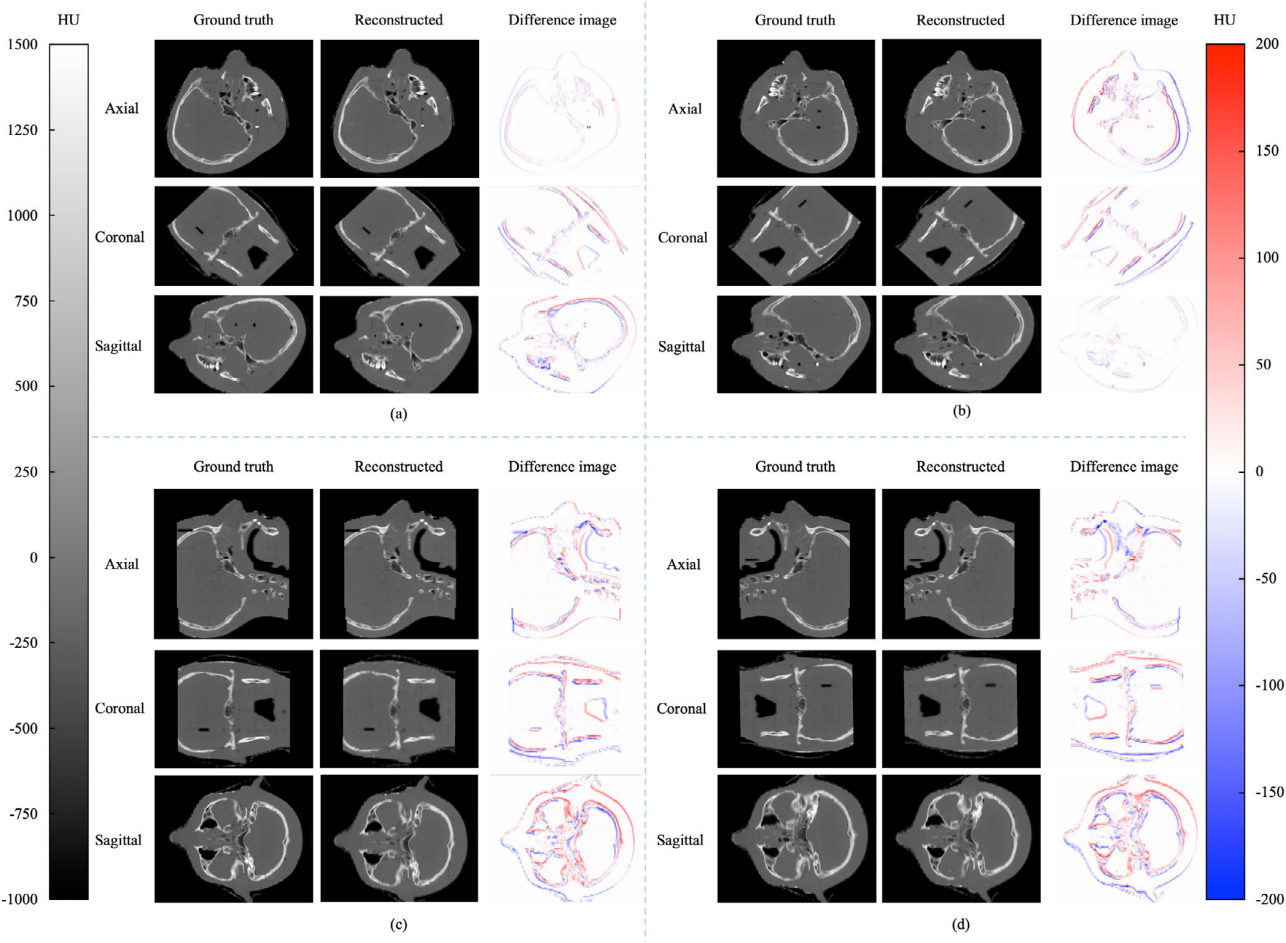
The reconstructed and ground truth central slices for phantom 1 in the axial, coronal, and sagittal planes for couch angles of (a) 45°, (b) −45°, (c) 90°, and (d) −90°. Color bars on both sides indicate the image display window/level.

**FIGURE 10:**
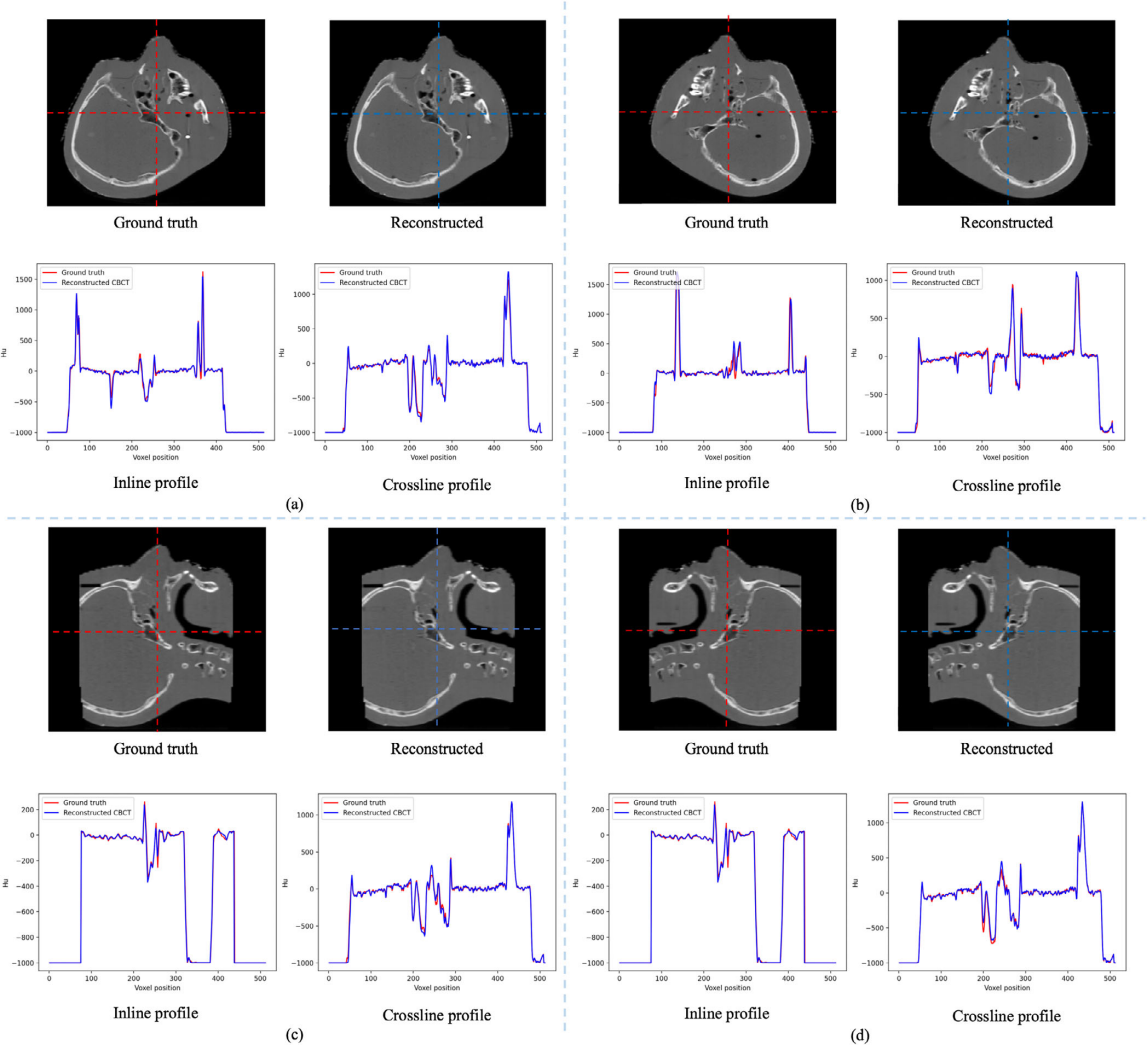
The inline and crossline of the axial plane of the central slice of the gCBCT and rCBCT images for phantom 1 for couch angles of (a) 45°, (b) −45°, (c) 90°, and (d) −90°. The positions of the inline and crossline profiles are marked by the red and blue dashed lines, respectively. rCBCT, reconstructed non‐coplanar CBCT.

The proposed method was also evaluated using two head phantoms. Figure 9 shows the central slices of the rCBCT and the gCBCT of phantom 1 in the axial, coronal, and sagittal planes in the cases of the four couch angles. The proposed GAN model improved the quality of rCBCT images at the couch angles of ±45° compared with the couch angles of ±90° at certain views. Figures 9 and 10 show that the proposed method reconstructed most of the structural information of phantom 1, including bones and teeth.

**FIGURE 11:**
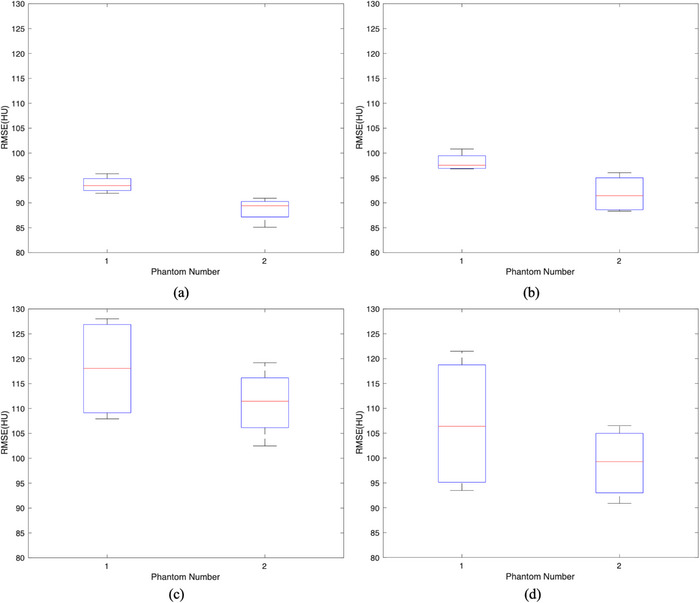
The RMSE between gCBCT and rCBCT images using phantom testing data for couch angles of (a) 45°, (b) −45°, (c) 90°, and (d) −90°. rCBCT, reconstructed non‐coplanar CBCT; RMSE, root mean square error.

**FIGURE 12:**
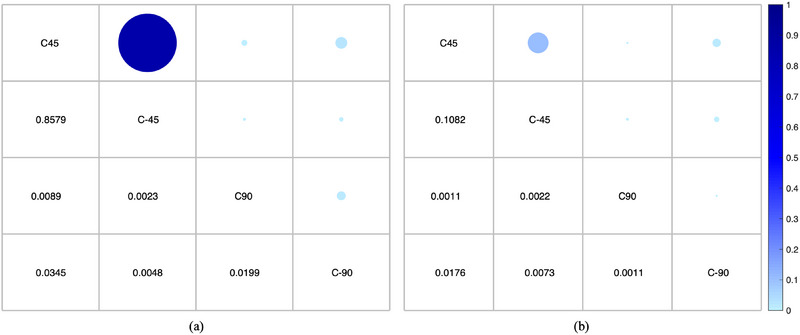
The *p*‐values of (a) the RMSE value and (b) the ε value compared multiple times under four couch angle groups in phantom data evaluation. C45, C‐45, C90, and C‐90 represent the couch angle of 45°, −45°, 90°, −90°, respectively. The larger the *p*‐value, the larger the corresponding blue circle and the darker the color. RMSE, root mean square error.

As shown in Figure 11, the mean RMSE values between the gCBCT and rCBCT images for couch angles ±45° are smaller than that for the couch angles ±90°. The *p*‐values show significant differences in RMSE values between the groups associated with the couch angles of ±45° and ±90°, as shown in Figure 12a. For the phantom data, the largest RMSE values were 95.85 HU, 100.81 HU, 128.01 HU, and 121.49 HU for couch angles of (a) 45°, (b) −45°, (c) 90°, and (d) −90°, respectively. The mean RMSE values were 91.19 HU, 95.01 HU, 114.58 HU, and 102.92 HU for the couch angles of (a) 45°, (b) −45°, (c) 90°, and (d) −90°, respectively.

**FIGURE 13:**
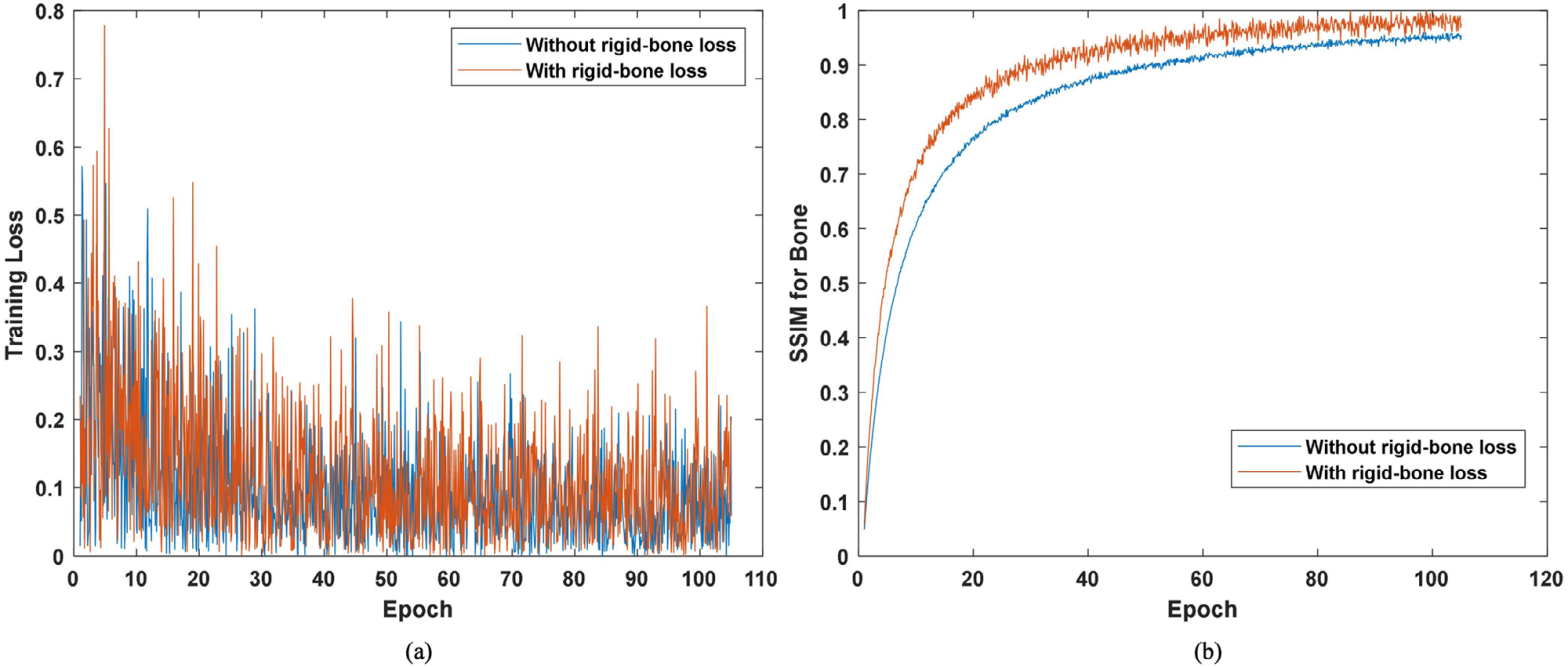
The values of (a) training loss and (b) SSIM during the training process.

**FIGURE 14:**
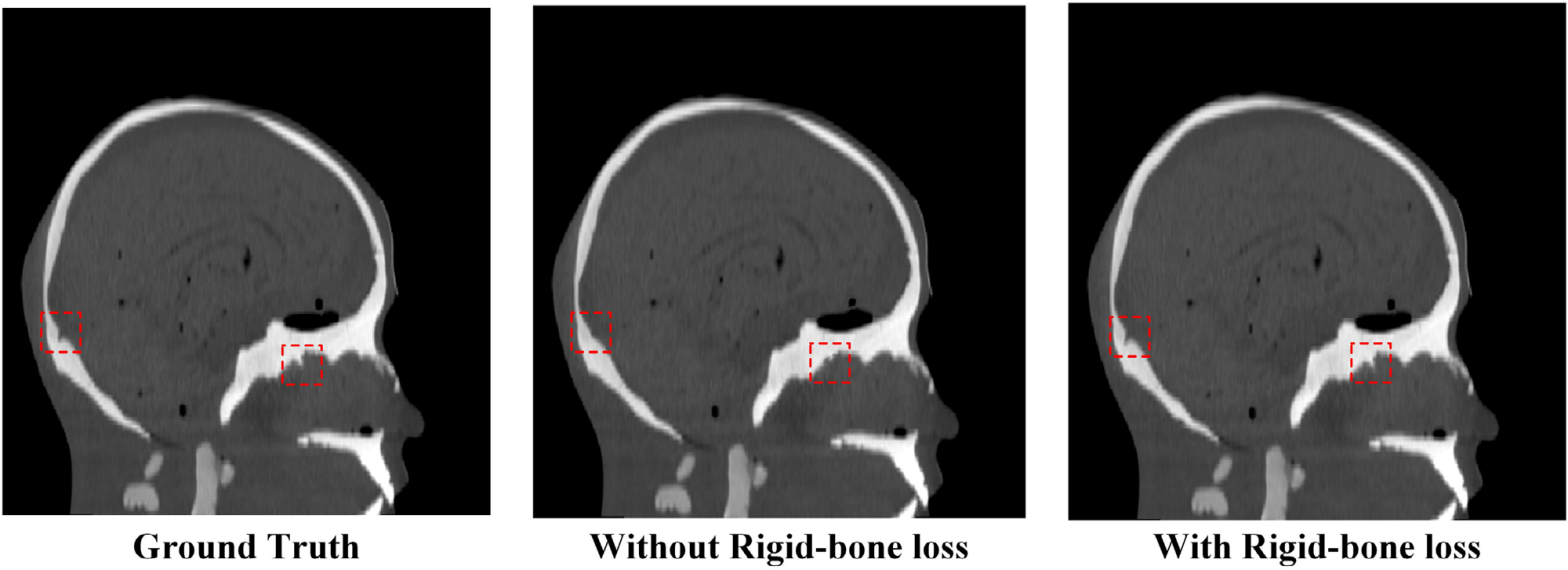
The ground truth as well as the reconstruction results with and without rigid‐bone loss of patient 1.

Table 3 summarizes the overall registration errors and the six transformation parameters of phantom data obtained at the four couch angles. Similar to the results of the patient data, the ε value for couch angle 90° was the largest, and all ε values were within 1 mm at all couch angles. Except for the no statistical difference between the couch 45° and couch −45° groups in the results of the *ε* values (*p* = 0.1082), there were significant differences associated with the *ε* values in the comparisons of other couch angle groups.

**TABLE 3:** The transform parameters (mean ± std) and overall registration error (mean ± std) between gCBCT and rCBCT of phantom testing data at four couch angles.

Couch angle (°)	$t_{x}$ (mm)	$t_{y}$ (mm)	$t_{z}$ (mm)	$r_{x}$ (°)	$r_{y}$ (°)	$r_{z}$ (°)	ε (mm)
45	0.130 (0.078)	0.186 (0.040)	0.238 (0.028)	0.295 (0.052)	0.192 (0.024)	0.283 (0.091)	0.457 (0.136)
−45	0.186 (0.084)	0.216 (0.017)	0.210 (0.035)	0.284 (0.067)	0.254 (0.030)	0.232 (0.077)	0.451 (0.151)
90	0.228 (0.109)	0.301 (0.022)	0.233 (0.059)	0.303 (0.080)	0.195 (0.097)	0.352 (0.088)	0.589 (0.169)
−90	0.225 (0.082)	0.248 (0.069)	0.285 (0.025)	0.261 (0.073)	0.226 (0.081)	0.275 (0.091)	0.517 (0.158)

## 4 DISCUSSION

In this study, we proposed a GAN model to reconstruct non‐coplanar CBCT images from coplanar CBCT images and non‐coplanar projections with a limited angle range for non‐coplanar radiotherapy. To the best of our acknowledge, this is the first study to reconstruct CBCT images in non‐coplanar situations using a deep‐learning model. The performance of the proposed model was evaluated using both patient simulation data and realistic phantom data at four couch angles. The reconstructed image quality was evaluated according to our qualitative and quantitative analyses, as shown in Figures 3, 4, 5, 6, 7, 8, 9, 10, 11, 12 and Tables 2 and 3.

As shown in Figures 8 and 12, groups with similar projection ranges, such as the couch 45° and couch −45°, have similar reconstruction performance and there are no statistical differences in the RMSE values and ε values. With an increasing number of non‐coplanar projections, the GAN model can provide more qualitatively and quantitatively improved results by preserving more details and recovering accurate tissue data, a difference that was significant between couch ± 45° and ± 90° groups (*p* < 0.05). This was consistent with the results reported by Meng et al.^16^ When the number of projections with couch angles of ±90° is increased by increasing the projection frequency, or by simultaneously acquiring MV projections that are converted to KV projections using a linear conversion function,^56^ the reconstruction quality of the image may be further improved.^17^, ^57^

For non‐coplanar radiotherapy, which often delivers higher fractional doses, couch rotation prolongs the setup time and increases the risk of movement during the treatment fraction.^1^, ^58^, ^59^ Therefore, intra‐treatment image guidance is critical to ensure anatomical accuracy before the next non‐coplanar beam radiation.^56^, ^60^ The proposed method can detect setup errors and maintain the anatomical structures in rCBCT images, which is helpful for intra‐treatment image guidance in non‐coplanar radiotherapy.

Although the proposed GAN model exhibits satisfactory results in non‐coplanar CBCT image reconstruction, there are still some limitations that must be noted. First, we only validated the performance of the phantom at four couch angles (±45° and ±90°) in this study. However, this model can be used for non‐coplanar CBCT image reconstruction at other couch angles. Others can modify the couch angle setting in the model to train their models according to their clinical needs and requirements. In addition, the proposed method can be potentially utilized for other treatment sites in non‐coplanar radiotherapy instead of being limited to brain cases. Second, each couch angle necessitates the training of a new GAN model, which inevitably increases the training costs. Training a model to handle arbitrary couch angles will be performed in our future studies. Third, the proposed model was not validated using data from actual patients, whose structures are more complex than those of the phantom. For clinical implementation, more clinical trials and clinical evaluations should be conducted to evaluate the effectiveness of the proposed model.

There are several manually determined parameters in the loss function that need manual determination. For both λ1 and λ2, values were set according to the experimental results. We have tried to adjust these two parameters, where λ1 ranged from 80 to 120 and λ2 ranged from 0.05 to 0.15, respectively. According to the values of RMSE and registration error, the proposed method achieved the best performance with λ1 being 100 and λ2 being 0.1. As for the function CM(·), we tried different values for the order k. It was shown that when the k increased from 1 to 4, there is a reduction in the values of RMSE. Thus, in this study, these parameters were set to be the optimal values for the best performance. We admitted if the dataset was very large, it may not be feasible to test all the combinations of different parameter values. For such circumstances, automatic hyperparameter tunning strategies, like random searching could be applied to find the optimal values.

## 5 CONCLUSIONS

In this study, we have proposed a non‐coplanar CBCT image reconstruction method using a GAN model. With the newly designed joint loss, the generator can generate non‐coplanar images that maintain the consistency of the global structure. The proposed method makes intra‐treatment image guidance possible in non‐coplanar radiotherapy.

## AUTHOR CONTRIBUTIONS

Ran Wei performed the model building, model training, and writing; Zhiyue Song performed the data collection, data analysis, and writing; Ziqi Pan, Ying Cao, and Yongli Song helped to collect data and writing; Jianrong Dai supervised the research activity planning and execution, and performed editing of the manuscript.

## CONFLICT OF INTEREST STATEMENT

The authors have no conflict of interest to disclose.

## ETHICS STATEMENT

This study was approved with exemption from informed consent by the independent ethics committee of Cancer Hospital, Chinese Academy of Medical Sciences.

## Data Availability

The data that support the findings of this study are available upon request from the authors.
